# Hypofractionated radiotherapy in ten fractions for postmastectomy patients: a phase II study compared with another hypofractionation schedule with sixteen fractions

**DOI:** 10.1186/s12885-021-09032-8

**Published:** 2021-12-01

**Authors:** Huayong Jiang, Lingling Meng, Huijuan Zhang, Xiangkun Dai, Qian Zhang, Zhongjian Ju, Wei Yu, Lin Ma

**Affiliations:** 1 Medical School of Chinese PLA, 28 Fuxing Road, Beijing, 100853 China; 2 Department of Radiation Oncology, Senior Department of Oncology, the Fifth Medical Center of PLA General Hospital, 100 West Fourth Ring Middle Road, Beijing, 100859 China

**Keywords:** Breast cancer, Hypofractionated radiotherapy, Postmastectomy

## Abstract

**Background:**

The purpose of this phase II study was to evaluate the feasibility of hypofractionated radiotherapy (HFRT) with a dose of 36.5 Gy in 10 fractions in postmastectomy patients.

**Methods:**

From March 2014 to December 2015, 85 patients with locally advanced breast cancer were eligible to participate in this study with a schedule of 36.5 Gy in 10 fractions. Intensity-modulated radiation therapy (IMRT) was delivered to the chest wall with or without the supraclavicular region. The primary endpoint was radiation-related toxicities. The secondary endpoints were locoregional failure-free survival (LRFFS), disease-free survival (DFS) and overall survival (OS). And the outcomes were compared with our retrospective study of 72 patients with 42.5 Gy in 16 fractions.

**Results:**

The median follow-up was 69.0 (range 66.5-71.5) months in the 36.5 Gy group and 93.0 (range 91.9-94.1) months in the 42.5 Gy group, respectively. Radiation-related toxicities were mainly grade 1, although a few patients had grade 2 plexopathy (1.2%) and acute skin toxicity (1.2%) in the 36.5 Gy group, and grade 2 acute skin toxicity (5.6%) and lymphedema (4.2%) in the 42.5 Gy group. There were no significant differences between the groups in acute and late toxicities. For all the patients, the 5-year LRFFS, DFS and OS were 97.7 and 100.0%, 93.1 and 90.3%, 98.8 and 97.2%, respectively, without significant differences between the groups.

**Conclusion:**

Postmastectomy HFRT with a schedule of 36.5 Gy in 10 fractions was feasible, with mild toxicities and excellent 5-year clinical outcome.

**Trial registration:**

Trial registration number: ChiCTR-ONRC-14004391.

Date of registration: 9/3/2014.

## Background

According to the latest estimates on the global burden of cancer released by the International Agency for Research on Cancer (IARC) in Dec 2020, female breast cancer is the most commonly occurring cancer worldwide (accounting for 11.7% of total new cases), posing a serious threat to women’s health [1]. In patients with locally advanced breast cancer, postmastectomy radiotherapy is an important part of multidisciplinary treatment and has been shown to significantly reduce the risk of locoregional recurrence and breast cancer mortality [2–4].

For breast cancer patients, conventionally fractionated radiotherapy (CFRT) typically delivers 50Gy in 25 fractions of 2Gy over 5 weeks. In recent years, hypofractionated radiotherapy (HFRT) as an alternative to CFRT has been widely carried out in patients treated with breast-conserving surgery [5–7]. Whereas, postmastectomy HFRT delivery has been limited because of the lack of high-level evidence, although data from a few published studies indicate that moderate HFRT seems feasible to deliver with an efficacy comparable to that of CFRT, for example, 43.5Gy in 15 fractions [8], 36.63Gy in 11 fractions [9], 23Gy in 4 fractions [10], and 40Gy in 15 fractions, 26 or 27Gy in 5 fractions [11].

Beginning in Mar 2014, we launched a monocentric phase II study with a novel schedule of 36.5Gy in 10 fractions, which is one of the shortest courses of postmastectomy HFRT designed to date. We retrospectively analysed the data of our previous study on HFRT with 42.5Gy in 16 fractions as the historical control to confirm the feasibility of the hypofractionation regimen with 10 fractions.

## Methods

### Study design and patients

This study is a prospective phase II trial (registration number ChiCTR-ONRC-14004391), with 85 patients recruited in a single centre (First Medical Centre of the Chinese PLA General Hospital) from March 2014 to December 2015. Another retrospective study on HFRT with the schedule of 42.5Gy in 16 fractions was conducted from April 2010 to September 2013, with 72 patients enrolled. The inclusion criteria of patients were as follows: Female patients with invasive breast cancer were eligible if they were aged 18-70 years, had a KPS of 70% or higher, had undergone mastectomy and axillary lymph node dissection with negative margins, had no reconstruction of the breast, had at least one pathologically positive axillary lymph node or a primary tumour with stage pT3-4 disease if they had undergone primary surgery (or a cT3-4 tumour or pathologically positive axillary lymph nodes if they had received chemotherapy before surgery), and had a timespan of less than 8 months between radiotherapy and mastectomy. Patients with positive hormone receptors were treated with hormonotherapy, and those with positive HER2 gene expression were treated with trastuzumab. The characteristics of the patients are shown in Table 1, without a significant difference between the groups (p > 0.05). This trial was approved by the ethics board of the Chinese PLA General Hospital, and all eligible patients provided written informed consent.

**Table 1:** Patient characteristics

	CW irradiation			CW + SR irradiation		
	42.5Gy/16F (n = 33)	36.5Gy/10F (n = 44)	***p***	42.5Gy/16F (n = 39)	36.5Gy/10F (n = 41)	***p***
**Age**			0.188			0.666
**< 50 years**	13 (39.4%)	24 (54.5%)		20 (51.3%)	23 (56.1%)	
**≥ 50 years**	20 (60.6%)	20 (45.5%)		19 (48.7%)	18 (43.9%)	
**TNM stage (AJCC 7.0)**^a^			0.322^#^			0.224^#^
**IIa**	20 (60.6%)	22 (50.0%)		2 (5.1%)	0	
**IIb**	13 (39.4%)	21 (47.7%)		2 (5.1%)	0	
**IIIa**	0	1 (2.3%)		25 (64.1%)	28 (68.3%)	
**IIIc**	0	0		10 (25.7%)	13 (31.7%)	
**Histological grade**			0.675^#^			0.955^#^
**I**	1 (3.1%)	2 (4.5%)		1 (2.6%)	1 (2.4%)	
**II**	21 (63.6%)	29 (65.9%)		23 (58.9%)	24 (58.5%)	
**III**	11 (33.3%)	13 (29.6%)		15 (38.5%)	16 (39.1%)	
**Laterality**			1.000			0.621
**Left**	18 (54.5%)	24 (54.5%)		24 (61.5%)	23 (56.1%)	
**Right**	15 (45.5%)	20 (45.5%)		15 (38.5%)	18 (43.9%)	
**ER-positive**	24 (63.9%)	28 (63.6%)	0.399	22 (57.9%)	30 (73.2%)	0.116
**PR-positive**	23 (69.7%)	27 (61.4%)	0.448	21 (53.8%)	28 (68.3%)	0.365
**HER2-positive**	6 (18.2%)	10 (22.7%)	0.627	8 (21.1%)	10 (24.4%)	0.678
**ER/PR/HER2-negative**	5 (15.2%)	11 (25.0%)	0.292	10 (26.3%)	4 (9.8%)	0.062
**LVSI-positive**	5 (15.2%)	6 (13.6%)	1.000^#^	7 (18.4%)	10 (24.4%)	0.481
**Chemotherapy**	32 (97.0%)	44 (100%)	0.429^#^	39 (100%)	41 (100%)	–
**Neoadjuvant CT**	0	0	–	10 (25.6%)	8 (19.5%)	0.512
**Adjuvant CT**	32 (97.0%)	44 (100%)	0.429^#^	29 (74.4%)	33 (80.5%)	0.512
**Hormonotherapy**	27 (81.8%)	29 (65.9%)	0.121	24 (61.5%)	30 (73.2%)	0.209
**Trastuzumab treatment**	6 (18.2%)	10 (22.7%)	0.627	8 (21.1%)	10 (24.4%)	0.678

*Abbreviations*: *CW* Chest wall, *SR* Supraclavicular region, *AJCC* American Joint Committee on Cancer, *ER* Estrogen receptor, *PR* Progesterone receptor, *LVSI* Lymphovascular space invasion, *CT* Chemotherapy. ^a^Pathologic stage of postmastectomy patients without neoadjuvant chemotherapy or prechemotherapy clinical stage for patients with neoadjuvant chemotherapy. ^#^ *p* value calculated by the Mann–Whitney U test

### Radiotherapy

All the patients were treated in a supine position and immobilised on a breast board. Computed tomography–based treatment planning was mandatory. The clinical target volume (CTV) included the ipsilateral chest wall for patients with pT3-4 tumours or one to three pathologically positive axillary lymph nodes, and included the chest wall and supraclavicular fossa (including the supraclavicular-infraclavicular area and level III axillary nodal region) for patients with four or more metastatic nodes or with pathologically positive nodes if they had received neoadjuvant chemotherapy. The planning target volume (PTV) was the CTV with an expansion of 5 mm while remaining 3 mm under the skin surface. Patients received step-and-shoot IMRT using 6-MV photon beams. The chest wall was irradiated with the 2- or 4-beam tangential IMRT technique, while 6-beam integrated IMRT plans were created to irradiate not only the chest wall but also the supraclavicular fossa. Radiotherapy was delivered five days per week, one fraction per day, without tissue-equivalent bolus application or mastectomy scar boost. Dose homogeneity within the treatment volume was required to be within 90 to 110% of the prescribed dose, and V105%-V107% < 5%, V107%-V110% < 2%.

For patients in the 36.5Gy group, the dose-volume constraints for organs at risk (OARs) were as follows: ipsilateral lung V16 < 20%, contralateral lung V5 < 8%; the mean dose of heart <5Gy for women with tumours in the left breast, and < 2.3Gy for those with tumours in the right breast. For patients in the 42.5Gy group, the dose-volume constraints were as follows: ipsilateral lung V20 < 20%, contralateral lung V5 < 10%; the mean dose of heart <5.5Gy for left breast cancer patients, and < 2.4Gy for right breast cancer patients. The dose to the contralateral breast should be as low as possible.

Patients underwent weekly physical examination during treatment and up to 2 weeks after radiotherapy, and then were followed up every three months for two years, every six months from 3 to 5 years, and yearly thereafter. Acute and late toxicities were evaluated according to the established Common Terminology Criteria for Adverse Events, version 4.0.

### Statistical analyses

The Mann–Whitney U test was used for the analysis of acute and late toxicities. Locoregional failure-free survival (LRFFS), disease-free survival (DFS) and overall survival (OS) were assessed by the Kaplan-Meier test. All data analyses were performed using the SPSS 24.0 (IBM Company, USA) statistical data package, and p < 0.05 was considered significant.

## Results

### Radiation-related toxicities

The incidences of acute and late toxicities in the two groups are shown in Table 2. No significant difference was detected for pneumonitis and dermatitis, as acute toxicity, in either group. One patient in the 36.5Gy group had grade 2 brachial plexopathy. No grade 3 or higher acute or late toxicities were observed, and no patients suffered from shoulder dysfunction or rib fractures in either group. There was no significant difference between the groups in the incidence of late toxicities.

**Table 2:** Adverse events

	36.5Gy/10F (n = 85)	42.5Gy/16F (n = 72)	***p***
**Acute toxicity**			
Skin toxicity			0.097
Grade 0	39 (45.9%)	27 (37.5%)	
Grade 1	45 (52.9%)	41 (56.9%)	
Grade 2	1 (1.2%)	4 (5.6%)	
Pneumonitis			0.496
Grade 0	51 (60.0%)	47 (65.3%)	
Grade 1	34 (40.0%)	25 (34.7%)	
**Late toxicity**			
Lung fibrosis			0.413
Grade 0	60 (70.6%)	55 (76.4%)	
Grade 1	25 (29.4%)	17 (23.6%)	
Skin toxicity			0.425
Grade 0	76 (89.4%)	67 (93.1%)	
Grade 1	9 (10.6%)	5 (6.9%)	
Ischemic heart disease			0.333
Grade 0	84 (98.8%)	69 (95.8%)	
Grade 1	1 (1.2%)	3 (4.2%)	
Lymphoedema			0.095
Grade 0	67 (78.8%)	59 (81.9%)	
Grade 1	18 (21.2%)	10 (13.9%)	
Grade 2	0	3 (4.2%)	
Brachial plexopathy			1.000
Grade 0	84 (98.8%)	72 (100%)	
Grade 1	0	0	
Grade 2	1 (1.2%)	0	

### Clinical outcomes

**Fig. 1:**
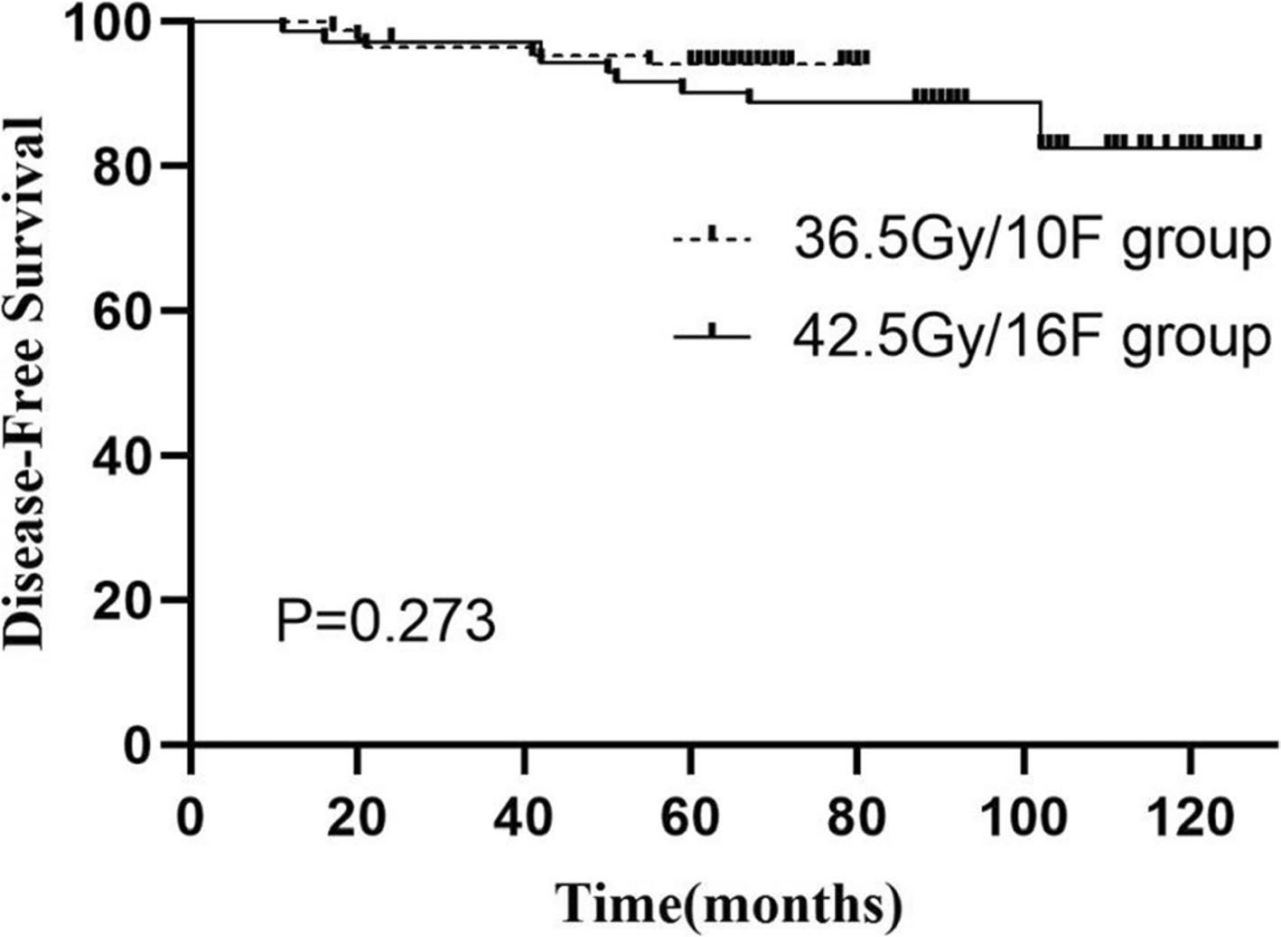
Disease-free survival as estimated by Kaplan-Meier analysis

**Fig. 2:**
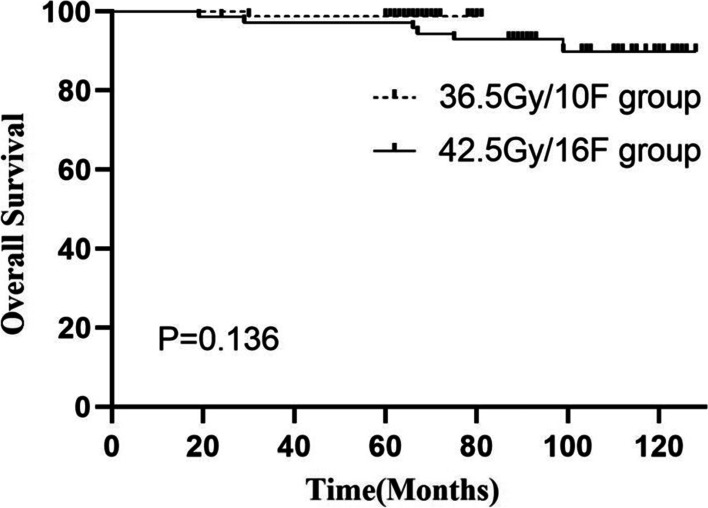
Overall survival as estimated by Kaplan-Meier analysis

At the time of last follow-up, one patient (pT2pN1, ER/PR/HER2-negative, with chest wall irradiation) in the 36.5Gy group developed regional recurrence (in the nonirradiated supraclavicular fossa), while no locoregional recurrence was detected in the 42.5Gy group. The 5-year LRFFS rates were 97.7 and 100.0% in the 36.5Gy and 42.5Gy groups, respectively. Four of 85 patients (4.7%) in the 36.5Gy group and 10 of 72 (13.9%) in the 42.5Gy group developed distant metastases, with 5-year DFS rates of 93.1 and 90.3%, respectively (Fig. 1). One patient (1.2%) and 6 (8.3%) died of breast cancer, with 5-year OS rates of 98.8 and 97.2% in the 36.5Gy and 42.5Gy groups, respectively (Fig. 2). No significant difference in 5-year LRFFS, DFS or OS was detected between the two groups (p = 0.194, 0.173 and 0.194, respectively).

## Discussion

At present, HFRT with a 3-week of 40-43.5 Gy is the commonest schedule, which has been confirmed the equivalence to 5-week of 50Gy for both efficacy and toxicity in breast cancer patients [5–8]. Currently, HFRT with 40-42.5 Gy in 3 weeks has been widely carried out in patients treated with breast-conserving surgery and is considered as an acceptable standard regimen [5–7]. In 2019, the first phase III trial of postmastectomy HFRT from China reported that the 3-week schedule of HFRT (43.5Gy/15F) was non-inferior to the standard 5-week schedule of CFRT (50Gy/25F) in patients with high-risk locally advanced breast cancer, with less grade 3 acute skin toxicity [8].

This study is a novel HFRT schedule of 36.5Gy in 10 fractions using IMRT technique for postmastectomy patients. According to radiobiological principles, the biologically effective dose (BED) of the HFRT schedule for breast cancer was about 70Gy, corresponding to an equivalent dose in 2Gy fractions (EQD2) of 49Gy, using linear quadratic formalism and an α/β ratio of 4Gy, an α value of 0.3, a T_pot_ value of 13 days, and an initial time lag of 14 days [12]. Compared to CFRT, HFRT with an increased fractional dose and lower total dose can protect normal tissue without compromising treatment efficacy. In addition, shortening the duration of radiotherapy to 2 weeks can effectively overcome the accelerated proliferation of breast cancer cells in the later course of CFRT [13, 14].

Data from our study indicated that the delivery of this postmastectomy HFRT regimen was feasible, with a very low incidence of grade 2 or more acute and late toxicities and good 5-year locoregional control. In the current study, acute and late toxicities were mainly grade 1. Compared with the study of Wang et al [8], in which the chest wall was irradiated using electron beam, and toxicity associated with both CFRT and HFRT was evaluated, acute skin toxicity in our study was mild, and no grade 3 or more toxicity was noted. Although the incidence of grade 1 pneumonitis was higher, there was no grade 2 toxicity. Late skin toxicity was also mild. The incidence of grade 1 toxicity was only about 10%. Although the incidence of grade 1 lung fibrosis was higher, there was no grade 2 toxicity. Other late toxicities were comparable or less severe. Grade 2 plexopathy was detected in one patient in May 2020, fifty-eight months after radiotherapy, in the 36.5Gy group. As the retrospective analysis of the patient’s treatment plan showed the maximum dose to the brachial plexus was 37.6Gy (0.2 cc), well below the dose-volume constraints of the nerve tissue, this case could be regarded as an incidental event, which has rarely been reported in moderate postmastectomy HFRT.

Results of radiation-related toxicities and clinical outcomes in the current study are consistent with a few published studies [7, 9–11, 15–18]. In the UK START-B trial with a median follow-up of 9.9 years, the 10-year locoregional relapse rate (4.3% vs 5.5%) did not differ significantly between the HFRT (40Gy/ 15F) and CFRT (50Gy/ 25F) groups. Although breast shrinkage, telangiectasia, and breast oedema were significantly less common normal tissue effects in the HFRT group, the proportion of postmastectomy patients was only 8.0% (177 patients) in the trial [7]. Khan et al [9] conducted a prospective phase II study with the delivery of 36.63Gy in 11 fractions to the chest wall or reconstructed breast and regional nodes with an optional 4-fraction mastectomy scar boost of 3.33Gy per fraction in 69 patients; no grade 3 toxicities were observed, and the 5-year update of the study showed no reported late grade 3 and 4 non-reconstruction-related toxicities, with a 5-year local control rate of 92% and an overall survival rate of 90% [15]. This trial, using 3-dimensional planning techniques, has a very similar hypofractionated schedule to ours. However, 97% of the patients received mastectomy scar boost, and 59% of patients had breast reconstruction in their trial, none in ours. Baillet et al [10] conducted a randomised study of CFRT (45Gy/ 25F/ 33d) versus a specific regimen of HFRT (23Gy/ 4F/ 17d, 5Gy of d1 and d3; 6.5Gy of d15 and d17) in patients with breast cancer. Preliminary analysis of the first 230 patients (35% of them had mastectomy) showed acceptable complications and locoregional recurrences in both groups with a minimum follow-up of 4 years. Kouloulias et al [16] retrospectively evaluated the efficacy and toxicity of two HFRT schedules (48.30Gy/ 21F and 42.56Gy/ 16F) compared to CFRT (45Gy/ 25F) in 117 postmastectomy patients. Although more grade 3 acute skin toxicity was noted in the HFRT groups than in the CFRT group (6.7 and 3.7% vs 0%), no significant difference was noted among the three groups in either acute or late toxicities after three years of follow-up. In another retrospective study, 980 patients treated with HFRT (2.65Gy/ F to a total of 42.4 to 53Gy) were compared to 660 patients treated with CFRT (2Gy/ F to a total of 50 to 60Gy). With a median follow-up of 71.8 months, HFRT showed higher grade 2 or more skin and subcutaneous toxicity but less lung and brachial plexus toxicity, without differences in 5-year LRRFS, DFS, and OS between the two schedules [17]. In the population-based analysis of a prospective provincial database containing 5487 patients in British Columbia (4006 patients with HFRT and 1481 patients with CFRT), for postoperative patients with lymph node-positive breast cancer treated with curative-intent breast or chest wall plus regional nodal irradiation (3152 patients had undergone mastectomy), no significant differences were observed in 10-year locoregional recurrence-free survival, distant recurrence-free survival, or breast cancer-specific survival between the HFRT (40 to 42.5Gy in 16 fractions) and CFRT (45 to 50.4Gy in 25 to 28 fractions) cohorts [18]. The shortest course of HFRT for breast cancer was reported by British scholar last year, the FAST-Forward multicentre phase III study, in which 4096 patients were randomly allocated to receive irradiation of the whole breast or chest wall with 40Gy in 15 fractions, 27Gy in 5 fractions or 26Gy in 5 fractions. There was no difference in disease control or adverse events between patients treated with the 1-week and the 3-week HFRT schedule. Although the 5-year incidence of ipsilateral breast tumour relapse was lower in the two HFRT groups, the proportion of postmastectomy patients was only 6.4% (193 patients) in study [11].

It is well known that the ipsilateral chest wall and regional lymphatic drainage areas are considered the primary target volume for postmastectomy radiotherapy. However, CTV delineation of the chest wall is controversial, especially in the definition of the ventral border. The RTOG guidelines define the chest wall as containing the skin and subcutaneous tissue [19]; however, the chest wall is defined as being 5 mm under the skin surface in the ESTRO guidelines [20]. Although the main location of chest wall relapse is the skin and subcutaneous tissue, a retrospective study showed that the ESTRO-CTV encompasses most locoregional recurrences [21]. At present, medical treatments for breast cancer have been intensified with the standard use of anthracyclines in chemotherapy, anti-HER2 targeted therapy, and long-term hormonotherapy, which might help to improve locoregional control in postmastectomy patients. In the study of Wang et al., only 55.3% of HER2-positive patients were treated with trastuzumab therapy, and the 5-year cumulative incidence of locoregional recurrence was greater than 8% [8]. In the current study, all patients who had not received standard medical treatments were excluded. With the PTV expanded from CTV remaining 3 mm below the skin surface and without tissue-equivalent bolus application or mastectomy scar boost, no chest wall recurrence was detected in either group.

In postmastectomy radiotherapy, irregular target volumes associated with the chest wall and regional nodes may increase the complexity of treatment planning, even when using a 3D conformal technique. IMRT has been increasingly used in breast cancer radiotherapy in recent years due to its excellent dose distribution [22]. Several dosimetric studies have demonstrated that IMRT provides better dose homogeneity and conformity to the planning target volume and a lower OAR irradiation dose than 2D-CRT and 3D-CRT [23–25]. IMRT shows an obvious dosimetric advantage when regional nodes and chest wall are assigned a complete PTV, for which 3D-CRT cannot meet critical OAR constraints [26]. The dosimetric advantages of IMRT are expected to ensure lower toxicity than conventional radiotherapy, and this property has been demonstrated in several prospective studies [27–30]. Lancellotta et al. showed the dosimetric advantage for treating the chest wall plus levels III-IV draining nodes after breast reconstruction compared with 3D conformal radiotherapy, linac-based IMRT and direct tomotherapy [29]. And their clinical study of 51 patients, irradiated by means of helical tomotherapy to the chest wall/breast plus draining nodes, showed that acute skin toxicity was mild and late skin toxicity was minimal [30]. In the current study, the acute and late side effects were mainly grade 1 toxicities, and few grade 2 and no grade 3 toxicities were noted. We believe that this result was partially due to the IMRT technique.

It is obvious that HFRT with fewer fractions and a shorter course is inherently more cost- and medical resource-effective for both patients and society. A rough calculation indicates that HFRT with a 2-week schedule instead of the 5-week CFRT might allow up to 60% more breast cancer patients to be treated with existing equipment, consequently results in an improvement in cancer-related survival, which has particular significance in countries with inadequate medical resources.

In conclusion, this study demonstrated a low incidence of radiation-related toxicities and satisfactory locoregional control for the schedule of postmastectomy HFRT with 36.5Gy in 10 fractions. Based on this study, we have designed a phase III trial (ChiCTR-2,100,042,855), in which we expect to confirm the noninferiority of the HFRT with 36.5Gy in 10 fractions to the CFRT with 50Gy in 25 fractions.

## Data Availability

The original datasets used and analysed during the current study are available from the corresponding author on reasonable request.

## Abbreviations

HFRT: Hypofractionated radiotherapy
CFRT: Conventional fractionated radiotherapy
IMRT: Intensity-modulated radiation therapy
LRFFS: Locoregional failure-free survival
DFS: Disease-free survival
OS: Overall survival
OAR: Organs at risk
CTV: Clinical target volume
PTV: Planning target volume
BED: Biologically effective dose
CW: Chest wall
SR: Supraclavicular region
AJCC: American Joint Committee on Cancer
ER: Estrogen receptor
PR: Progesterone receptor
LVSI: Lymphovascular space invasion
CT: chemotherapy
IARC: International Agency for Research on Cancer
